# Predicting Colorectal Cancer Survival Using Time-to-Event Machine Learning: Retrospective Cohort Study

**DOI:** 10.2196/44417

**Published:** 2023-10-26

**Authors:** Xulin Yang, Hang Qiu, Liya Wang, Xiaodong Wang

**Affiliations:** 1 School of Computer Science and Engineering University of Electronic Science and Technology of China Chengdu China; 2 Big Data Research Center University of Electronic Science and Technology of China Chengdu China; 3 Department of Gastrointestinal Surgery, West China Hospital Sichuan University Chengdu China

**Keywords:** colorectal cancer, survival prediction, machine learning, time-to-event, SHAP, SHapley Additive exPlanations

## Abstract

**Background:**

Machine learning (ML) methods have shown great potential in predicting colorectal cancer (CRC) survival. However, the ML models introduced thus far have mainly focused on binary outcomes and have not considered the time-to-event nature of this type of modeling.

**Objective:**

This study aims to evaluate the performance of ML approaches for modeling time-to-event survival data and develop transparent models for predicting CRC-specific survival.

**Methods:**

The data set used in this retrospective cohort study contains information on patients who were newly diagnosed with CRC between December 28, 2012, and December 27, 2019, at West China Hospital, Sichuan University. We assessed the performance of 6 representative ML models, including random survival forest (RSF), gradient boosting machine (GBM), DeepSurv, DeepHit, neural net-extended time-dependent Cox (or Cox-Time), and neural multitask logistic regression (N-MTLR) in predicting CRC-specific survival. Multiple imputation by chained equations method was applied to handle missing values in variables. Multivariable analysis and clinical experience were used to select significant features associated with CRC survival. Model performance was evaluated in stratified 5-fold cross-validation repeated 5 times by using the time-dependent concordance index, integrated Brier score, calibration curves, and decision curves. The SHapley Additive exPlanations method was applied to calculate feature importance.

**Results:**

A total of 2157 patients with CRC were included in this study. Among the 6 time-to-event ML models, the DeepHit model exhibited the best discriminative ability (time-dependent concordance index 0.789, 95% CI 0.779-0.799) and the RSF model produced better-calibrated survival estimates (integrated Brier score 0.096, 95% CI 0.094-0.099), but these are not statistically significant. Additionally, the RSF, GBM, DeepSurv, Cox-Time, and N-MTLR models have comparable predictive accuracy to the Cox Proportional Hazards model in terms of discrimination and calibration. The calibration curves showed that all the ML models exhibited good 5-year survival calibration. The decision curves for CRC-specific survival at 5 years showed that all the ML models, especially RSF, had higher net benefits than default strategies of treating all or no patients at a range of clinically reasonable risk thresholds. The SHapley Additive exPlanations method revealed that R0 resection, tumor-node-metastasis staging, and the number of positive lymph nodes were important factors for 5-year CRC-specific survival.

**Conclusions:**

This study showed the potential of applying time-to-event ML predictive algorithms to help predict CRC-specific survival. The RSF, GBM, Cox-Time, and N-MTLR algorithms could provide nonparametric alternatives to the Cox Proportional Hazards model in estimating the survival probability of patients with CRC. The transparent time-to-event ML models help clinicians to more accurately predict the survival rate for these patients and improve patient outcomes by enabling personalized treatment plans that are informed by explainable ML models.

## Introduction

Colorectal cancer (CRC) is the third most commonly diagnosed cancer and the second leading cause of cancer death worldwide, with 1.9 million new cases and 0.93 million deaths estimated in 2020, accounting for 10% of the global cancer incidence and 9.4% of all cancer-caused deaths [1,2]. With high morbidity and mortality, CRC is an important component of health care expenditure and imposes a heavy burden on families and society [3]. Precise survival prediction for patients with CRC will help clinicians optimize treatment measures, improve survival rates, and reduce the disease burden of patients [3,4]. Therefore, obtaining precise survival predictions for patients with CRC and understanding what affects these predictions are critical for identifying targeted interventions in the clinical setting.

The Cox proportional hazards (CPH) model [5] is the most commonly used statistical method for survival analysis, which has been widely applied to predict prognosis for patients with CRC due to its ease of use and interpretation [6,7]. To deal with high-dimensional data, based on the basic CPH model, some variant models of CPH were proposed, such as Lasso-Cox [8], EN-Cox [9], and robust CPH with nonlinearities and interactions [6]. In recent years, machine learning (ML), especially ensemble learning and deep learning (DL), has proven to be a great complement to traditional statistical methods in many health care applications [10-12]. A large body of studies has attempted to use ML models to predict CRC survival [4,13,14]. For instance, Pourhoseingholi et al [4] compared the performance of traditional and ensemble ML models for predicting the 5-year survival of patients with CRC. The results showed that the ensemble voting model achieved an area under the receiver operating characteristic curve of 0.96, which was the best result. Al-Bahrani et al [14] used deep neural networks to predict 1-year, 2-year, and 5-year survival for patients with CRC. The deep neural networks model achieved an average area under the receiver operating characteristic curve of 0.87, which is higher than the 0.85 reported by Stojadinovic et al [15].

Although ML-based approaches have shown great potential in CRC survival prediction, the vast majority of existing studies did not include time-to-event data and have only considered binary outcomes, which may incur the risk of bias in prediction accuracy [16,17]. Some time-to-event ML models, such as random survival forest (RSF) [18], gradient boosting machine (GBM) [19], DeepSurv [20], DeepHit [21], neural net-extended time-dependent Cox model (Cox-Time) [22], and neural multitask logistic regression (N-MTLR) [23], have shown promising performances in several prognostic studies on breast cancer [10,24], oral cavity cancer [16], and lung cancer [11,23]; however, it is not clear whether these models have the same advantages in CRC survival prediction. Moreover, due to the “black box” nature of ML models, the predictions made by these models are opaque, meaning that the importance of input features to the output is unclear, which limits the clinical applications of ML approaches. Therefore, it is essential to adopt effective methods to increase the transparency of ML models in the medical domain.

Given the high incidence of CRC and the lack of a reliable study on modeling time-to-event survival data of CRC using ML-based approaches, this study seeks to contribute to the existing body of knowledge by evaluating the performance of time-to-event ML models in predicting CRC-specific survival and by combining ML models with the SHapley Additive exPlanations (SHAP) method [25] to provide transparent predictions for clinical application.

## Methods

### Data Collection

We collected data from patients with CRC from the Database of Colorectal Cancer (DACCA) of West China Hospital, Sichuan University. This database includes patient demographics, diagnosis, tumor, treatment, and follow-up information. Specifically, the features collected included age at diagnosis, gender, marriage, BMI, operation time, preoperative carcinoembryonic antigen (CEA), number of positive lymph nodes (PLNs), dystrophy, obstruction, intussusception, intestinal perforation, diabetes, hypertension, differentiation, tumor-node-metastasis (TNM) staging based on the 8th edition of American Joint Committee on Cancer (TNM staging), morphologic type, histologic type, R0 resection, neoadjuvant treatment, cardiac function, anemia, perineural invasion, and tumor location.

The date of the last follow-up for this study was October 11, 2021. CRC cases were identified by the *International Classification of Diseases, Tenth Revision* codes (C18, C19, and C20). After discharge, the clinician would follow up with the patient regularly according to the patient’s condition and record the survival information. The inclusion criteria were as follows: (1) aged 15-99 years; (2) first diagnosed with CRC between December 28, 2012, and December 27, 2019; and (3) follow-up time ≥1 month.

### Ethics Approval

This study was approved by the Ethics Committee of West China Hospital, Sichuan University (2021-155). Because this study was a retrospective study design and all data were analyzed anonymously, the requirement to obtain informed consent was removed.

### Study Outcomes

The outcome of this study was CRC-specific survival, which was defined as the number of months from diagnosis to death from CRC or the end of follow-up, whichever occurred first.

### Data Preprocessing and Feature Selection

Features with a missing ratio of more than 30% were excluded [26,27] because they provided limited information. Missing data were assumed missing at random and were imputed 5 times in the package “miceforest” [28] by multiple imputation by chained equations, which helps minimize bias. The imputation model contained all candidate predictor variables. Imputations were performed within the cross-validation loop, and we developed an imputation model on the training set and used it to impute missing values on the training and testing sets, respectively. Because the dimensions were different, numerical features, ordinal categorical features, and nominal categorical features were processed using zero-mean normalization, integer encoding, and one-hot encoding, respectively.

We performed feature selection by combining the results of 2 different approaches: one based on the algorithm and the other based on clinical experience. For the algorithm-based approach, we used multivariate Cox regression to select features significantly associated with CRC-specific survival [16]. Features with *P* values <.05 were considered significantly associated with survival. For the clinical experience–based approach, clinical experts identified 6 features (age, preoperative CEA, PLN, TNM staging, R0 resection, and neoadjuvant treatment) as the most relevant to CRC-specific survival based on their clinical experience. The final feature set was the union of the feature sets selected by the above 2 approaches. We aimed to develop parsimonious models that contain only relevant and easily accessible features, appropriately preventing models from overfitting [29].

### Model Development

A total of 6 time-to-event ML models with 2 based on ensemble learning (RSF and GBM) and 4 based on DL (DeepSurv, DeepHit, Cox-Time, and N-MTLR) were developed to predict CRC-specific survival. These models were selected according to their promising performances reported in previous studies [16,23,30].

RSF is an ensemble learning algorithm similar to bagging [31], which consists of survival trees [18]. RSF grows survival trees by randomly selecting features and then splits nodes using candidate features to maximize the survival difference between child nodes. GBM is a gradient boosting–based ensemble learning algorithm consisting of base learners. GBM sequentially builds base learners in a greedy stage-wise fashion to minimize the weighted risk function [19]. DeepSurv is a DL-based algorithm that extends CPH to handle nonlinear effects between input features and clinical events. DeepSurv consists of multiple hidden layers and is trained with modern techniques, such as batch normalization and gradient descent optimization algorithms [20]. DeepHit is a DL-based nonproportional hazards algorithm that uses multitask learning to handle competition between events. DeepHit consists of a shared subnetwork and 1 or more cause-specific subnetworks [21]. Cox-Time is a DL-based algorithm that treats time as a regular covariate to model interactions between time and the other covariates. N-MTLR is a DL-based algorithm that builds different neural networks on different time intervals to estimate the probability of the event of interest occurring in each interval. RSF, GBM, DeepHit, Cox-Time, and N-MTLR algorithms have no proportional hazards assumption. To explore the difference in performance between the time-to-event ML model and the CPH model, we developed a robust CPH model with nonlinearities and interactions based on a study by Hippisley-Cox and Coupland [6]. A sample size calculation was performed using the “pmsampsize” [32] package in R for the CPH model, and the total required sample size was 555 patients. In each cross-validation loop, we had 1725 patients in the training set, meaning that our sample size was sufficient for modeling a reliable CPH model.

To tune all the time-to-event ML models’ hyper-parameters, we performed a Bayesian search [33] with stratified 5-fold cross-validation in the training set. The hyper-parameter search space of the ML models is shown in Multimedia Appendix 1.

### Evaluation of Model Performance

The discriminative ability of models was evaluated by the time-dependent concordance index (C^td^) [34], which is the ratio of correctly distinguished pairs to all pairs. A C^td^ value of 1 represents perfect discrimination, whereas a value of 0.5 represents random guessing. The Brier score [35] measures the distance between a patient’s survival status and the predicted probability of survival. The integrated Brier score (IBS) is the integral of the Brier score at all available times. The calibration ability of models was evaluated with IBS, where the smaller the IBS value of the model, the better its calibration ability. Additionally, we assessed the calibration of 5-year CRC-specific survival by comparing the observed survival probability at 5 years with the predicted survival probability.

Decision curve analysis is a statistical method to evaluate whether a model has utility in supporting clinical decisions by calculating the net benefit at different threshold probabilities [27]. Therefore, we used decision curve analysis to evaluate the net benefits of models for CRC survival at 5 years at a range of clinically reasonable risk thresholds (10%-30%) [36].

**Figure 1:**
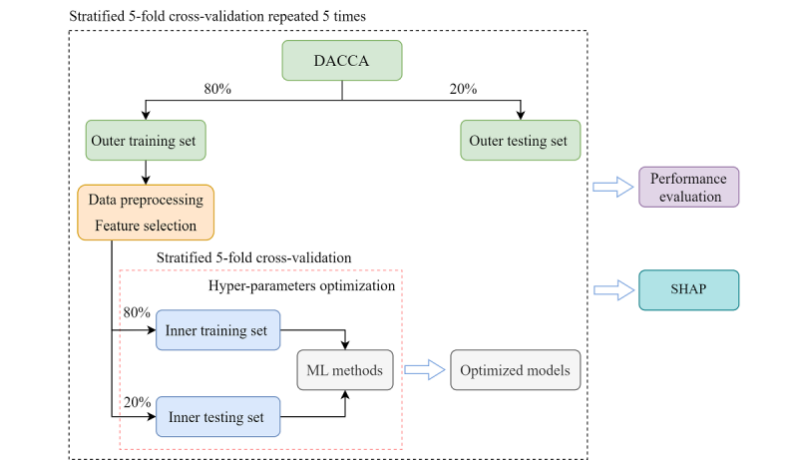
Study design flowchart.

All models were evaluated in stratified 5-fold cross-validation [37] repeated 5 times, as shown in Figure 1. During the inner stratified 5-fold cross-validation loop, we trained time-to-event ML models with different hyperparameter configurations on the inner training set and calculated their C^td^ on the inner testing set. The configuration that yielded the highest average C^td^ was chosen as the best hyperparameter configuration. During the outer 5 times stratified 5-fold cross-validation loop, the performance of the optimized time-to-event ML models was estimated on the outer testing data.

### Model Explanation

Model transparency is critical to the application of models in the medical domain. Therefore, to make time-to-event ML models more transparent, we introduced SHAP, which is a model-agnostic post hoc explanation algorithm that has been widely applied to explain ML models [10,38,39].

The 5-year survival is a metric commonly used in medical science to evaluate the effects of surgery and treatment. Thus, we adopted SHAP to explore important factors affecting 5-year CRC-specific survival. In this study, all testing data were selected to calculate the SHA*P* value of each feature to obtain the importance ranking of features.

### Sensitivity Analysis

Sensitivity analyses were performed to examine the predictive stability of the models for different subgroups. Model performance was evaluated in the subgroups, focusing on patients in different age groups (<65 years and ≥65 years) [40] and patients of different sex.

### Statistical Analysis

Categorical and Boolean features were presented as frequencies and percentages, and numerical features were presented as the median (25th and 75th percentiles). A Wilcoxon rank sum test was used to assess the difference in performance between the models. A 2-sided *P* value <.05 was considered statistically significant.

All analyses and calculations were performed using R (version 4.2.2; R Core Team) and Python (version 3.8.7; Python Software Foundation). This study followed the Guidelines for Developing and Reporting Machine Learning Predictive Models in Biomedical Research [41] and the Transparent Reporting of a Multivariable Prediction Model for Individual Prognosis or Diagnosis [42] statement.

## Results

### Patient Characteristics

A total of 2157 patients were included in this study. Statistical descriptions of these patients are presented in Multimedia Appendix 2. The median age of the 2157 patients was 61 years, and 1301 (60.4%) patients were male. Tumors were completely resected (R0 resection) in 1503 (69.6%) patients, and the median operation time was 60 minutes. Tumor pathology in very few patients was characterized by squamous cell carcinoma, and tumors were moderately differentiated in 1388 (64.3%) patients. These patients had a median preoperative CEA of 3.8 ng/mL, and 1217 (56.4%) patients received neoadjuvant treatment. Follow-up durations ranged from 1 month to 104 months. During this period, 420 (19.5%) patients died from CRC, 36 (1.6%) patients died from other causes, and 1702 (78.9%) patients survived during follow-up.

### Model Performance

The evaluation results of the time-to-event models are shown in Table 1. Among the 6 time-to-event ML models, the average C^td^ (0.789, 95% CI 0.779-0.799) of the DeepHit model is the highest and the average IBS (0.096, 95% CI 0.094-0.099) of the RSF model is the lowest, but these are not statistically significant (Multimedia Appendices 3 and 4). Additionally, no significant performance differences were observed between the RSF, GBM, DeepSurv, Cox-Time, and N-MTLR models and the CPH model for C^td^ and IBS (Multimedia Appendix 5).

**Table 1:** Performance of time-to-event models.

Model	Time-dependent concordance index, mean (95% CI)	Integrated Brier score, mean (95% CI)
Cox proportional hazards	0.781 (0.771-0.791)	0.098 (0.095-0.100)
Random survival forest	0.786 (0.776-0.796)	0.096 (0.094-0.099)
Gradient boosting machine	0.787 (0.775-0.799)	0.100 (0.097-0.102)
DeepSurv	0.787 (0.776-0.798)	0.097 (0.095-0.100)
DeepHit	0.789 (0.779-0.799)	0.108 (0.101-0.114)
Cox-Time^a^	0.787 (0.776-0.798)	0.097 (0.095-0.099)
Neural multitask logistic regression	0.786 (0.776-0.796)	0.098 (0.086-0.101)

^a^Neural net-extended time-dependent Cox.

**Figure 2:**
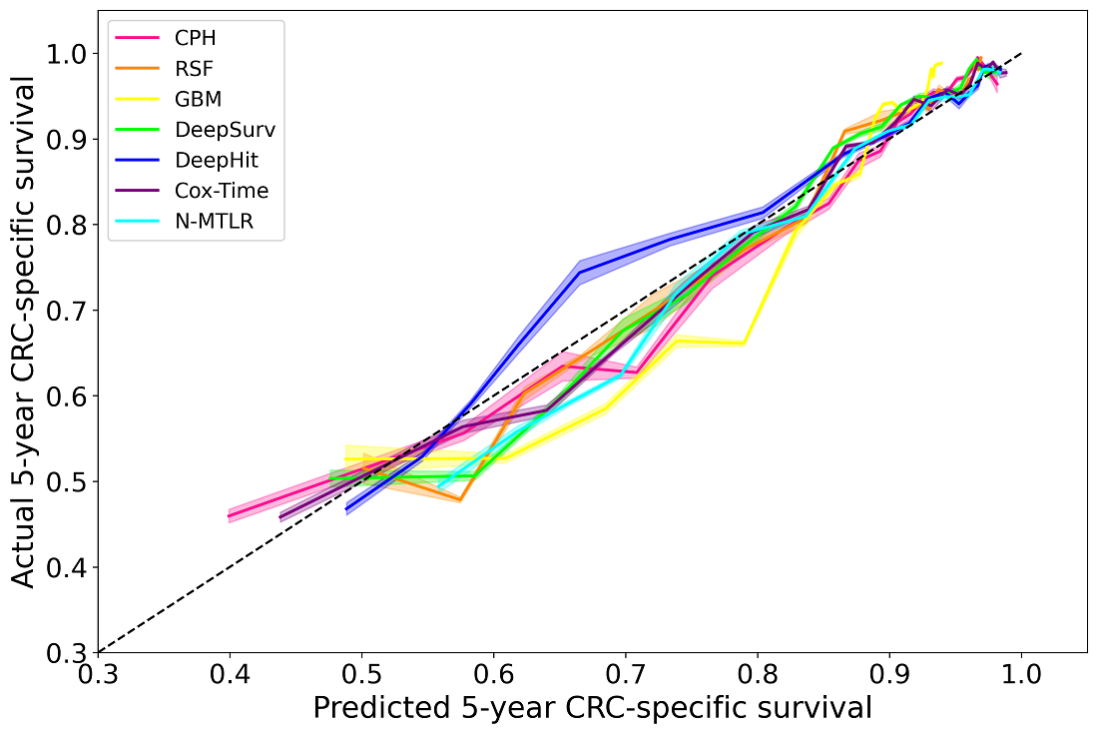
5-year colorectal cancer (CRC)–specific survival calibration plot. Cox-Time: neural net-extended time-dependent Cox; CPH:Cox proportional hazards; GBM: gradient boosting machine; N-MTLR: neural multitask logistic regression; RSF: random survival forest.

Figure 2 shows the difference between the predicted 5-year CRC-specific survival and the actual events. Overall, all models exhibited good 5-year survival calibration. The CPH, RSF, GBM, DeepSurv, Cox-Time, and N-MTLR models slightly overestimated the 5-year survival rate, while the DeepHit model slightly underestimated the 5-year survival rate. In addition, the CPH, RSF, DeepSurv, Cox-Time, and N-MTLR models produced better 5-year survival calibrations than the DeepHit and GBM models.

**Figure 3:**
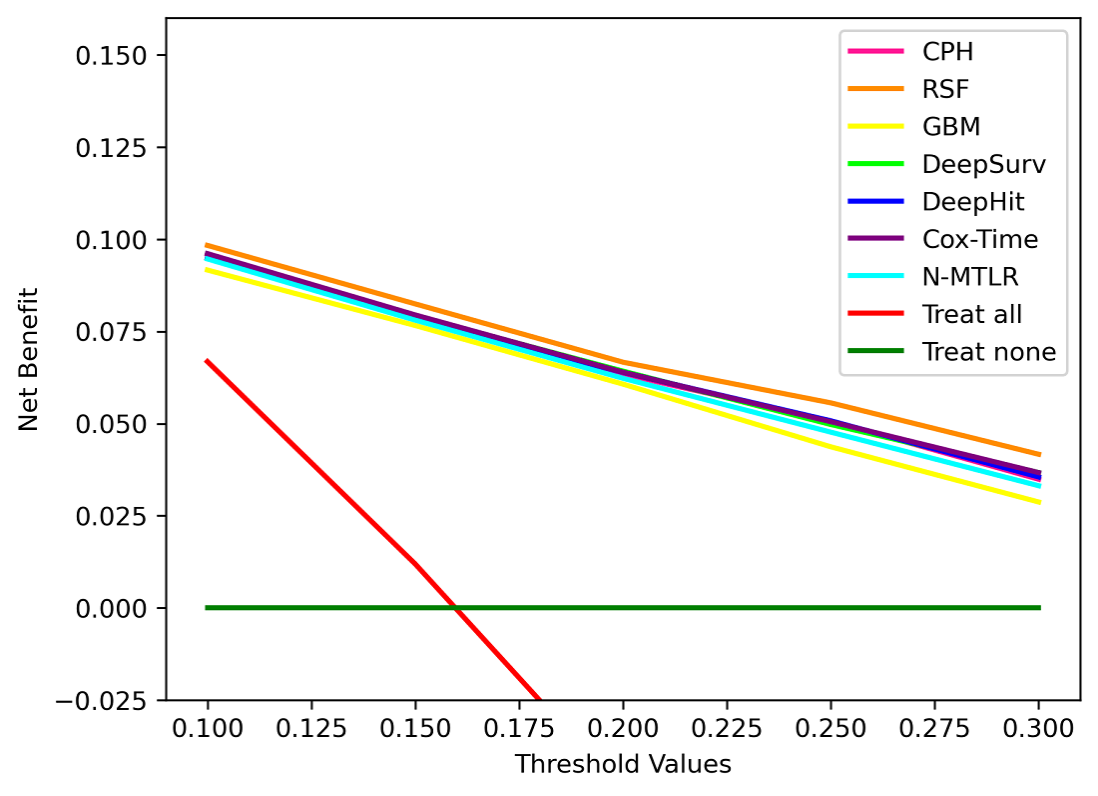
Decision curves for 5-year colorectal cancer (CRC)–specific survival. Cox-Time: neural net-extended time-dependent Cox; CPH: Cox proportional hazards; GBM: gradient boosting machine; N-MTLR: neural multitask logistic regression; RSF: random survival forest.

Figure 3 displays the net benefit curves for CRC survival models at 5 years. Overall, all the CRC survival models had higher net benefits than the default strategies of treating all or no patients at a range of clinically reasonable risk thresholds. In particular, the net benefit of the RSF model surpassed all other models.

### Feature Importance

**Figure 4:**
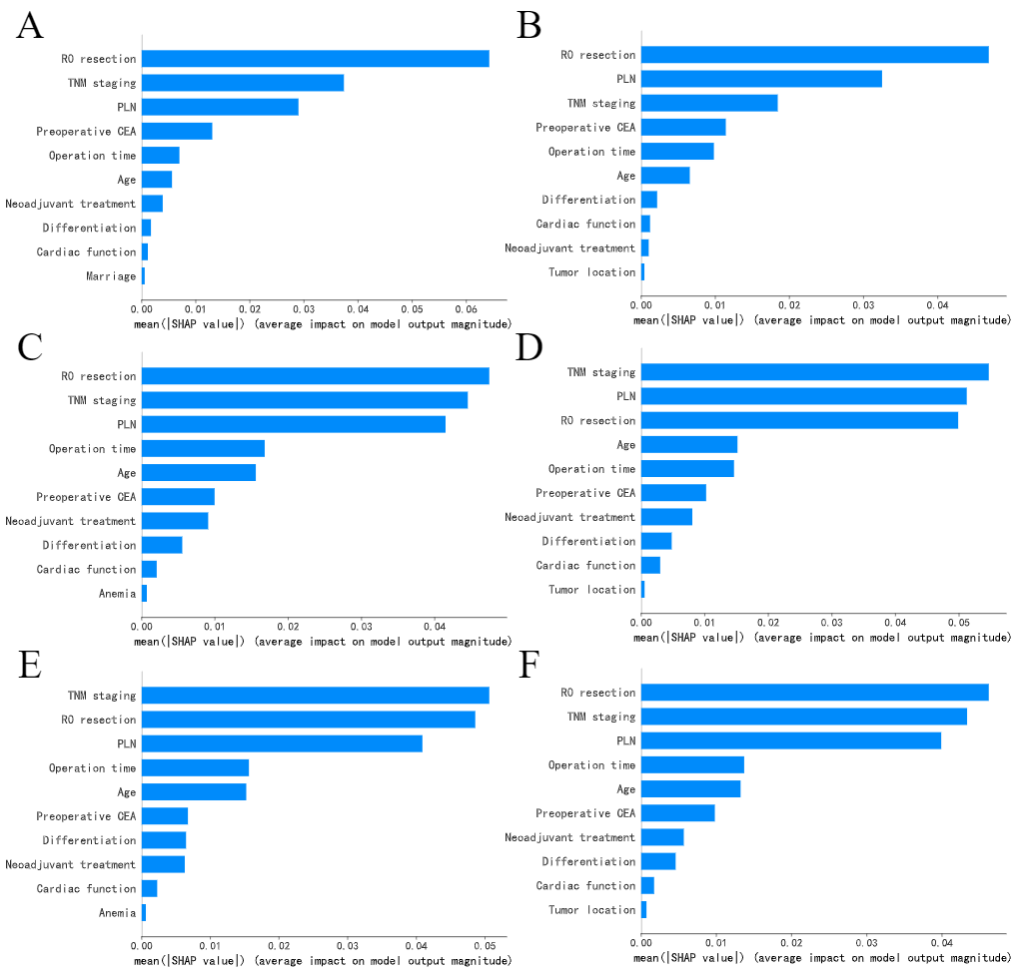
The importance ranking of the top 10 features for (A) random survival forest, (B) gradient boosting machine, (C) DeepSurv, (D) DeepHit, (E) neural net-extended time-dependent Cox (Cox-Time), and (F) neural multitask logistic regression according to the mean SHapley Additive exPlanation (SHAP) absolute value. CEA: carcinoembryonic antigen; PLN: positive lymph node; TNM: tumor-node-metastasis.

We applied SHAP to determine the effect of the input features on the 5-year CRC-specific survival. Figure 4 shows the importance ranking of the input features. The features are listed in a top-down order, with decreasing importance. The larger the mean SHAP absolute value of a feature, the more important that feature is. R0 resection, TNM staging, and PLN ranked among the top 3 in feature importance ranking for all ML models.

### Sensitivity Analysis

The C^td^ and IBS of the GBM and DeepHit models remained stable in different age and sex groups (Multimedia Appendix 6). The performance of the GBM, DeepSurv, DeepHit, and Cox-Time models has no statistical difference in different age stratifications, while the IBS of the CPH, RSF, and N-MTLR models in the age group ≥65 years is significantly lower than that in the age group <65 years. The performance of the GBM and DeepHit models has no statistical difference in different sex stratifications, while the IBS of the CPH, RSF, DeepSurv, Cox-Time, and N-MTLR models for female individuals is significantly lower than that for male individuals.

## Discussion

### Principal Findings

In this study, we evaluated the performance of traditional (CPH) and ML-based (RSF, GBM, DeepSurv, DeepHit, Cox-Time, and N-MTLR) models for CRC-specific survival prediction and applied SHAP to make predictions of time-to-event ML models more transparent. We found that the DeepHit model demonstrated the best discriminative ability (C^td^ 0.789, 95% CI 0.779-0.799) and the RSF model produced better-calibrated survival estimates (IBS 0.096, 95% CI 0.094-0.099), but these are not statistically significant. Moreover, the RSF, GBM, DeepSurv, Cox-Time, and N-MTLR models have comparable predictive accuracy to the CPH model in terms of discrimination and calibration. The 5-year CRC-specific survival calibration plot showed all the ML models exhibited good calibration. Decision curves for 5-year CRC-specific survival showed that all the ML models had higher net benefits than the default strategies of treating all or no patients, and the RSF model had the highest net benefit. Similar results have been reported in other cancer survival studies. For example, Du et al [43] used several models, including CPH and RSF, to predict disease-specific survival in patients with oral and pharyngeal cancers. Their results showed that time-to-event ML algorithms, such as RSF, provide nonparametric alternatives to CPH to estimate the survival probability of patients with oral and pharyngeal cancers. Adeoye al [16] found that RSF, DeepSurv, DeepHit, and Cox-Time algorithms are successful in predicting oral cavity cancer prognosis. Our results showed the potential of applying time-to-event ML predictive algorithms to help predict CRC-specific survival, and the RSF, GBM, Cox-Time, and N-MTLR nonproportional hazards algorithms could be used as nonparametric alternatives to CPH in CRC-specific survival prediction. Inconsistent with some previous studies [10,11,24], we did not find that the time-to-event ML models achieve better performance than the CPH model for CRC-specific prediction. One possible reason may be that the sample size of our data set is not large enough. ML approaches are data-driven approaches and may require truly “big data” to ensure their developed models avoid overfitting and their potential advantages (dealing with nonlinear relations and interactions) reach fruition [32]. Our data set is sufficient to develop a reliable CPH model, but larger sample sizes may be required when developing ML models. Another possible reason is that we only included a small number of features. The advantage of ML over traditional statistical methods is that it automatically deals with the interactions between numerous features based on data [44]. Therefore, if the number of features is too small, the advantage of ML will not be significant.

The results of the sensitivity analysis showed that the performance of the GBM and DeepHit models remained stable in different age and sex groups, while other models performed better in the age group ≥65 years and the female group. This may be related to a higher incidence of CRC deaths among individuals aged ≥65 years compared to those aged <65 years. This data set is unbalanced, so higher event (death from CRC) rates may lead to better performance. The proportion of female patients aged ≥65 years is higher than that of male patients, which may be one of the reasons why the models perform better in the female subgroup.

To the best of our knowledge, this is the first study to evaluate the discriminative ability and calibration ability of various time-to-event ML models trained with clinical features to predict CRC-specific survival based on data from Chinese patients with CRC. Censoring is an unavoidable problem in long-term survival prediction because patients are often lost to follow-up or die from unrelated causes. Although ML has been widely used in CRC survival prediction, many ML-based models ignore censoring because the default framework is to analyze binary outcomes rather than time-to-event survival outcomes, which may bias survival predictions. Time-to-event algorithms achieve a dynamic perception of survival predictions by providing estimates at various time points, and these algorithms can be better used for the survival monitoring of patients with CRC. However, how different ML-based time-to-event algorithms perform in terms of CRC-specific survival remains to be explored. The results of our study will fill this gap and provide a reference for subsequent researchers.

The predictions of ML models are opaque due to their “black box” nature. In this study, we used SHAP to make time-to-event ML models more transparent. SHAP is a model-agnostic ex post facto explanation method. The larger the SHA*P* value of a feature, the more influential it is on the model output. The visualization of feature importance showed that TNM staging, PLN, and preoperative CEA were important in predicting 5-year CRC-specific survival, which was consistent with those of previous works [3,4,6] and clinical experience. Additionally, we found R0 resection and operation time were important features in our study, which were rarely reported in the previous CRC literature. One possible reason for this result is that our model is based on data from Chinese patients with CRC, and it suggests that R0 resection and operation time may simply be valid independent predictors of CRC-specific survival in Chinese populations, suggesting that the features affecting the prediction of CRC-specific survival are different in different populations. The value of R0 resection and operation time in predicting CRC-specific survival is worthy of Chinese clinicians’ attention.

### Limitations

This study has some limitations. First, the retrospective nature of this study resulted in some overly missing features, such as perineural invasion. However, the features available for modeling produced satisfactory and reasonable estimates on the test set. Second, the information collected in this study is structured clinical data; if combined with structured clinical data and unstructured clinical data, such as imaging and multiomics data, it may provide better prediction results. Third, as with other cancer survival studies [6,10,17], unbalanced survival data sets were not processed. Last, the time-to-event ML models were trained on single-center CRC survival data and need to be further validated in external data sets.

### Conclusions

This study showed the potential of applying time-to-event ML predictive algorithms to help predict CRC-specific survival. The RSF, GBM, Cox-Time, and N-MTLR algorithms could provide nonparametric alternatives to CPH in estimating the survival probability of CRC patients. The transparent time-to-event ML models help clinicians more accurately predict the survival rate for patients with CRC and improve patient outcomes by enabling personalized treatment plans that are informed by explainable ML models.

## Appendix

Multimedia Appendix 1 The hyperparameter search space of machine learning models.

Multimedia Appendix 2 Features collected from the Database of Colorectal Cancer.

Multimedia Appendix 3 Wilcoxon rank sum test for the time-dependent concordance index between the DeepHit model and other models.

Multimedia Appendix 4 Wilcoxon rank sum test for the integrated Brier score between the random survival forest model and other models.

Multimedia Appendix 5 Wilcoxon rank sum test for pairwise comparison between the Cox proportional hazards model and other models.

Multimedia Appendix 6 Performance in stratified subgroups.

## Abbreviations

CEA: carcinoembryonic antigen
Cox-Time: neural net-extended time-dependent Cox
CPH: Cox proportional hazards
CRC: colorectal cancer
C^td^: time-dependent concordance index
DL: deep learning
GBM: gradient boosting machine
IBS: integrated Brier score
ML: machine learning
N-MTLR: neural multitask logistic regression
PLN: positive lymph node
RSF: random survival forest
SHAP: SHapley Additive exPlanation
TNM: tumor-node-metastasis

## Data Availability

The data sets analyzed during this study are not publicly available due to the personal information protection requirement of the ethics committee but are available from the corresponding author on reasonable request.
