# Supplementary material for: Predicting Colorectal Cancer Survival Using Time-to-Event Machine Learning: Retrospective Cohort Study
Source: J Med Internet Res. 2023 Oct 26;25:e44417. doi: 10.2196/44417 (PMC10636616; doi:10.2196/44417)
Supplement: Multimedia Appendix 1 [file jmir_v25i1e44417_app1.doc]

**Appendix 1.** The hyper-parameters search space of ML models.

| **Model hyper-parameter** | | **Hyper-parameter search space** |
| --- | --- | --- |
| **RSF** | |  |
|  | n_estimators | Logarithmic space ranging from 25 to 750 |
|  | max_depth | Logarithmic space ranging from 1 to 25 |
|  | min_sample_split | Logarithmic space ranging from 5 to 50 |
|  | min_sample_leaf | Logarithmic space ranging from 1 to 50 |
| **GBM** | |  |
|  | n_estimators | Logarithmic space ranging from 25 to 750 |
|  | max_depth | Logarithmic space ranging from 1 to 25 |
|  | min_sample_split | Logarithmic space ranging from 5 to 50 |
|  | min_sample_leaf | Logarithmic space ranging from 1 to 50 |
|  | learning_rate | Logarithmic space ranging from 0.0001 to 0.1 |
|  | dropout | {0.0,0.2,0.3,0.4,0.5,0.6,0.7,0.8} |
|  | subsample | {0.2,0.3,0.4,0.5,0.6,0.7,0.8,0.9,1.0} |
| **DeepSurv** | |  |
|  | learning_rate | Logarithmic space ranging from 0.0001 to 0.1 |
|  | batch_size | {64,128,256,512} |
|  | dropout | {0.0,0.2,0.3,0.4,0.5,0.6,0.7,0.8} |
|  | num_layer | {1,2,3,4,5} |
|  | num_node | Logarithmic space ranging from 8 to 256 |
|  | epoch | {10,20,30,40,50,60,70,80,90,100} |
|  | optimzer | {SGD, Adam, AdamW, AdamWR} |
|  | activate_func | {SELU,Sigmoid,Relu,Relu6,Tanh,LeakyRelu} |
| **DeepHit** | |  |
|  | learning_rate | Logarithmic space ranging from 0.0001 to 0.1 |
|  | batch_size | {64,128,256,512} |
|  | dropout | {0.0,0.2,0.3,0.4,0.5,0.6,0.7,0.8} |
|  | num_layer | {1,2,3,4,5} |
|  | num_node | Logarithmic space ranging from 8 to 256 |
|  | epoch | {10,20,30,40,50,60,70,80,90,100} |
|  | optimzer | {SGD, Adam, AdamW, AdamWR} |
|  | activate_func | {SELU,Sigmoid,Relu,Relu6,Tanh,LeakyRelu} |
|  | sigma | {0.1,0.2,0.3,0.4,0.5,0.6,0.7,0.8,0.9,  1,2,3,4,5,6,7,8,9,10} |
|  | alpha | {0.0,0.1,0.2,0.3,0.4,0.5,0.6,0.7,0.8,0.9,1.0} |
|  | num_duration | {10,15,30,50} |
| **Cox-Time** | |  |
|  | learning_rate | Logarithmic space ranging from 0.0001 to 0.1 |
|  | batch_size | {64,128,256,512} |
|  | dropout | {0.0,0.2,0.3,0.4,0.5,0.6,0.7,0.8} |
|  | num_layer | {1,2,3,4,5} |
|  | num_node | Logarithmic space ranging from 8 to 256 |
|  | epoch | {10,20,30,40,50,60,70,80,90,100} |
|  | optimzer | {SGD, Adam, AdamW, AdamWR} |
|  | activate_func | {SELU,Sigmoid,Relu,Relu6,Tanh,LeakyRelu} |
| **N-MTLR** | |  |
|  | learning_rate | Logarithmic space ranging from 0.0001 to 0.1 |
|  | batch_size | {64,128,256,512} |
|  | dropout | {0.0,0.2,0.3,0.4,0.5,0.6,0.7,0.8} |
|  | num_layer | {1,2,3,4,5} |
|  | num_node | Logarithmic space ranging from 8 to 256 |
|  | epoch | {10,20,30,40,50,60,70,80,90,100} |
|  | optimzer | {SGD, Adam, AdamW, AdamWR} |
|  | activate_func | {SELU,Sigmoid,Relu,Relu6,Tanh,LeakyRelu} |
|  | num_duration | {10,15,30,50} |
