# Supplementary material for: Predicting Colorectal Cancer Survival Using Time-to-Event Machine Learning: Retrospective Cohort Study
Source: J Med Internet Res. 2023 Oct 26;25:e44417. doi: 10.2196/44417 (PMC10636616; doi:10.2196/44417)
Supplement: Multimedia Appendix 2 [file jmir_v25i1e44417_app2.doc]

**Appendix 2. Features collected from the DACCA.**

| **Feature** | | **N = 2157** |
| --- | --- | --- |
| Age, mean (SD) |  | 61.0 (51.0-68.0) |
| Body mass index, mean (SD) |  | 22.8 (20.7-25.1) |
| Operation time, mean (SD) |  | 60 (50-80) |
| Preoperative CEA, mean (SD) |  | 3.8 (2.0-9.3) |
| PLN, mean (SD) |  | 0 (0-2) |
| **Gender, n (%)** | |  |
|  | female | 856 (39.6) |
|  | male | 1301 (60.4) |
| **Marriage, n (%)** | |  |
|  | Unmarried | 28 (1.2) |
|  | Married | 2129 (98.8) |
| **Dystrophy, n (%)** | |  |
|  | Yes | 513 (23.7) |
|  | No | 1356 (32.3) |
| **Obstruction, n (%)** | |  |
|  | No obstruction | 754 (34.9) |
|  | incomplete obstruction | 945 (43.8) |
|  | complete obstruction | 202 (9.3) |
| **Intussusception, n (%)** | |  |
|  | Yes | 96 (4.4) |
|  | No | 1788 (82.8) |
| **Intestinal perforation, n (%)** | |  |
|  | Yes | 44 (2.0) |
|  | No | 1839 (85.2) |
| **Diabetes, n (%)** | |  |
|  | Yes | 252 (11.6) |
|  | No | 1506 (69.8) |
| **Hypertension, n (%)** | |  |
|  | Yes | 576 (26.7) |
|  | No | 1231 (57.0) |
| **Differentiation, n (%)** | |  |
|  | Poor | 607 (28.1) |
|  | Moderate | 1388 (64.3) |
|  | Well | 39 (1.8) |
| **TNM staging, n (%)** | |  |
|  | Ⅰ | 243 (11.4) |
|  | Ⅱ | 494 (22.9) |
|  | Ⅲ | 618 (28.6) |
|  | Ⅳ | 571 (26.4) |
| **Morphologic type, n (%)** | |  |
|  | Massive | 178 (8.2) |
|  | Polypoid | 63 (2.9) |
|  | Ulceration | 1650 (76.4) |
|  | Elevated | 248 (11.4) |
| **Histologic type, n (%)** | |  |
|  | Adenocarcinoma | 1713 (79.4) |
|  | Mucus adenocarcinoma | 332 (15.3) |
|  | Signet-ring cell carcinoma | 43 (1.9) |
|  | Neuroendocrine carcinoma | 16 (0.7) |
|  | Squamous cell carcinoma | 14 (0.6) |
|  | Other types | 16 (0.7) |
| **R0 resection, n (%)** | |  |
|  | Yes | 1503 (69.6) |
|  | No | 434 (20.1) |
| **Neoadjuvant treatment, n (%)** | |  |
|  | Yes | 1217 (56.4) |
|  | No | 782 (36.2) |
| **Cardiac function, n (%)** | |  |
|  | Normal | 1610 (74.6) |
|  | Ⅰ | 223 (10.3) |
|  | Ⅱ | 13 (0.6) |
| **Anemia, n (%)** | |  |
|  | Yes | 1110 (51.4) |
|  | No | 777 (36.0) |
| **Perineural invasion, n (%)** | |  |
|  | Yes | 96 (4.4) |
|  | No | 0 (0.0) |
| **Tumor location, n (%)** | |  |
|  | Colon | 464 (21.5) |
|  | Rectum | 1690 (78.3) |
| Follow-up time, mean (SD) |  | 52 (35-73) |
| **Survival state, n (%)** | |  |
|  | Survival | 1701 (78.9) |
|  | Died of colorectal cancer | 420 (19.5) |
|  | Died of other causes | 36 (1.6) |
