# Supplementary material for: Predicting Colorectal Cancer Survival Using Time-to-Event Machine Learning: Retrospective Cohort Study
Source: J Med Internet Res. 2023 Oct 26;25:e44417. doi: 10.2196/44417 (PMC10636616; doi:10.2196/44417)
Supplement: Multimedia Appendix 3 [file jmir_v25i1e44417_app3.doc]

**Appendix 3.** Wilcoxon rank sum test for C^td^ between the DeepHit model and other models.

| Model | *P* value |
| --- | --- |
| CPH | .27 |
| RSF | .62 |
| GBM | .78 |
| DeepSurv | .88 |
| Cox-Time | .90 |
| N-MTLR | .64 |
