# Supplementary material for: Predicting Colorectal Cancer Survival Using Time-to-Event Machine Learning: Retrospective Cohort Study
Source: J Med Internet Res. 2023 Oct 26;25:e44417. doi: 10.2196/44417 (PMC10636616; doi:10.2196/44417)
Supplement: Multimedia Appendix 4 [file jmir_v25i1e44417_app4.doc]

**Appendix 4.** Wilcoxon rank sum test for IBS between the RSF model and other models.

| Model | *P* value |
| --- | --- |
| CPH | .65 |
| GBM | .07 |
| DeepSurv | .65 |
| DeepHit | .01 |
| Cox-Time | .78 |
| N-MTLR | .23 |
