# Supplementary material for: Predicting Colorectal Cancer Survival Using Time-to-Event Machine Learning: Retrospective Cohort Study
Source: J Med Internet Res. 2023 Oct 26;25:e44417. doi: 10.2196/44417 (PMC10636616; doi:10.2196/44417)
Supplement: Multimedia Appendix 5 [file jmir_v25i1e44417_app5.doc]

**Appendix 5.** Wilcoxon rank sum test for pairwise comparison between the CPH model and other models.

| Model | *P* value | |
| --- | --- | --- |
|  | C^td^ | IBS |
| RSF | .54 | .65 |
| GBM | .48 | .17 |
| DeepSurv | .36 | .96 |
| DeepHit | .27 | .02 |
| Cox-Time | .37 | .71 |
| N-MTLR | .62 | .39 |
