# Supplementary material for: Predicting Colorectal Cancer Survival Using Time-to-Event Machine Learning: Retrospective Cohort Study
Source: J Med Internet Res. 2023 Oct 26;25:e44417. doi: 10.2196/44417 (PMC10636616; doi:10.2196/44417)
Supplement: Multimedia Appendix 6 [file jmir_v25i1e44417_app6.doc]

**Appendix 6.** Model performance and Wilcoxon rank sum test results in stratified subgroups.

| Model performance | | Age <65 | Age≥65 | Female | Male | *P* value | |
| --- | --- | --- | --- | --- | --- | --- | --- |
|  | |  |  |  |  | Age <65 vs Age≥65 | Female vs Male |
| **CPH** | |  |  |  |  |  |  |
|  | C^td^ | 0.779 | 0.790 | 0.797 | 0.775 | .27 | .03 |
|  | IBS | 0.100 | 0.094 | 0.095 | 0.100 | .04 | .006 |
| **RSF** | |  |  |  |  |  |  |
|  | C^td^ | 0.778 | 0.801 | 0.797 | 0.778 | .01 | .12 |
|  | IBS | 0.099 | 0.092 | 0.094 | 0.099 | .003 | .02 |
| **GBM** | |  |  |  |  |  |  |
|  | C^td^ | 0.784 | 0.791 | 0.798 | 0.780 | .42 | .14 |
|  | IBS | 0.101 | 0.097 | 0.097 | 0.102 | .08 | .05 |
| **DeepSurv** | |  |  |  |  |  |  |
|  | C^td^ | 0.786 | 0.793 | 0.797 | 0.780 | .49 | .13 |
|  | IBS | 0.099 | 0.094 | 0.095 | 0.100 | .05 | .02 |
| **DeepHit** | |  |  |  |  |  |  |
|  | C^td^ | 0.788 | 0.795 | 0.800 | 0.783 | .25 | .05 |
|  | IBS | 0.110 | 0.104 | 0.106 | 0.109 | .05 | .22 |
| **Cox-Time** | |  |  |  |  |  |  |
|  | C^td^ | 0.785 | 0.794 | 0.797 | 0.781 | .28 | .15 |
|  | IBS | 0.099 | 0.094 | 0.095 | 0.099 | .05 | .01 |
| **N-MTLR** | |  |  |  |  |  |  |
|  | C^td^ | 0.785 | 0.792 | 0.796 | 0.780 | .37 | .11 |
|  | IBS | 0.100 | 0.095 | 0.096 | 0.100 | .03 | .03 |
